# Exploring the Effects of a Mediterranean Diet and Weight Loss on the Gut Microbiome and Cognitive Performance in Older, African American Obese Adults: A Post Hoc Analysis

**DOI:** 10.3390/nu15153332

**Published:** 2023-07-27

**Authors:** Andrew McLeod, Beatriz Penalver Bernabe, Yinglin Xia, Jennifer Sanchez-Flack, Melissa Lamar, Linda Schiffer, Nefertiti Oji-Njideka Hemphill, Giamila Fantuzzi, Pauline Maki, Marian Fitzgibbon, Lisa Tussing-Humphreys

**Affiliations:** 1 Department of Kinesiology and Nutrition, University of Illinois Chicago, Chicago, IL 60612, USA; giamila@uic.edu (G.F.); ltussing@uic.edu (L.T.-H.); 2 Institute for Health Research and Policy, University of Illinois Chicago, Chicago, IL 60608, USA; jsanch38@uic.edu (J.S.-F.); lschiff@uic.edu (L.S.); mlf@uic.edu (M.F.); 3 Department of Biomedical Engineering, University of Illinois Chicago, Chicago, IL 60612, USA; penalver@uic.edu; 4 Department of Medicine, University of Illinois Chicago, Chicago, IL 60612, USA; yxia@uic.edu (Y.X.); melissa_lamar@rush.edu (M.L.); 5 Department of Pediatrics, University of Illinois Chicago, Chicago, IL 60612, USA; 6 University of Illinois Cancer Center, University of Illinois Chicago, Chicago, IL 60612, USA; 7 Rush Alzheimer’s Disease Center, Rush University, Chicago, IL 60612, USA; 8 Nutrient Innovation LLC, Chicago, IL 60607, USA; nefertiti@nutrientinnovation.com; 9 Departments of Psychology and Psychiatry, University of Illinois Chicago, Chicago, IL 60612, USA; pmaki1@uic.edu

**Keywords:** gut microbiota, cognition, Mediterranean diet, African Americans, Alzheimer’s dementia

## Abstract

African American adults have a higher prevalence of Alzheimer’s dementia (AD) than non-Hispanic Whites. The impact of a Mediterranean Diet (Med Diet) and intentional weight loss (IWL) on the gut microbiome may alter AD risk. A post hoc analysis of the Building Research in Diet and Cognition (BRIDGE) trial was performed to determine whether participation in an 8-month Med Diet lifestyle intervention with (*n* = 35) or without IWL (*n* = 31) was associated with changes in gut microbiota structure, abundance, and function and whether these changes were related to changes in cognitive performance. The results showed that family and genus alpha diversity increased significantly in both groups combined (*p* = 0.0075 and *p* = 0.024, respectively). However, there were no other significant microbially related within- or between-group changes over time. Also, an increase in Med Diet adherence was significantly associated with a decrease in alpha diversity at the phylum level only (*p* = 0.049). Increasing alpha diversity was associated with decreasing cognitive performance, but this association was attenuated after controlling for Med Diet adherence. In sum, an 8-month Med Diet lifestyle intervention with or without IWL did not appreciably alter the gut microbiome.

## 1. Introduction

In 2022, one in nine individuals over the age of 65 in the United States had Alzheimer’s dementia (AD), a type of dementia caused by Alzheimer’s disease (ADx) [1]. By 2060, that proportion is expected to grow to one in seven older adults [2,3]. Moreover, African American adults have a significantly higher 2020 US Census-adjusted prevalence of AD than non-Hispanic Whites and Hispanics [3]. This disparity may be due in part to the higher prevalence of risk factors for AD in African American adults compared with White adults, including higher rates of obesity [4,5] and lower diet quality [6,7]. Efforts to reduce the incidence of AD have centered on detecting AD and ADx earlier, in the prodromal phase called mild cognitive impairment (MCI); half of patients with this condition progress to AD [1]. Because efficacious treatment for AD remains elusive [8], primary prevention through identifying and mitigating risk factors is critical, as it may prevent or delay up to 37% of cases of ADx in the United States [4].

Two such risk factors are poor diet and excess body weight. A poor diet, such as the Western Diet [9]—characterized by high consumption of saturated fatty acids, refined grains, processed meats, and added sugars—is associated with increased biomarkers of AD. For example, in middle-aged adults with intact cognition, a Western Diet, compared with a Med Diet, was associated with higher levels of amyloid-beta, a protein that coalesces to form plaques in the brain and contributes to neuronal death and symptoms of AD [1,10]. Furthermore, in a recent randomized controlled trial, a 4-week Western Diet in cognitively intact middle-aged adults decreased cerebral perfusion, which is associated with an increased risk of AD [11,12]. Regarding excess body weight, of the eight modifiable risk factors for dementia identified by several consensus groups, such as the World Health Organization, midlife obesity was ranked number one, contributing to nearly 18% of all cases of AD and related dementias in the U.S. [4]. Moreover, in one study, those in the top quintile of waist circumference at midlife compared with those in the lowest quintile had an almost threefold increased risk of dementia an average of 36 years later (hazard ratio = 2.72, 95% confidence interval (CI) = 2.33–3.33) [13].

Several studies have concluded that consuming a Mediterranean Diet (Med Diet), consisting of whole grains, vegetables, fruits, olive oil, and red wine, and intentional weight loss (IWL) are associated with protection from AD and improved cognitive performance [7,14,15,16]. Regarding the Med Diet, a meta-analysis of five longitudinal studies (*n* = 6111) showed that for those highly adherent to a Med Diet, the risk of AD was almost half of the risk for those who had low adherence (relative risk = 0.6, 95% CI = 0.48–0.77) [7]. Some of the proposed mechanisms that link a Med Diet to neuroprotection are its ability to reduce inflammation and oxidative stress [17,18] and improve cognitive performance [19], all of which are risk factors for AD [1,20,21,22]. In regard to excess body weight, the most recent meta-analysis showed that in both longitudinal studies and randomized controlled trials (RCTs), IWL among obese and overweight adults leads to improved cognitive performance [16]. Specifically, in the RCTs, compared with controls, those who lost on average 2 kg/m^2^ of BMI through diet or physical activity had better attention (*n* = 326, standardized mean difference (SMD) = 0.44, 95% CI = 0.26–0.62), memory (*n* = 349, SMD = 0.35, 95% CI = 0.12–0.57), and language use (*n* = 326, SMD = 0.21, 95% CI = 0.05–0.37) [16]. IWL may improve cognitive performance by reducing systemic inflammation and oxidative stress [23].

Another mechanism by which a Med Diet and IWL may improve cognition is the gut microbiome. A Med Diet is associated with higher microbial diversity and increased fecal anti-inflammatory microbial metabolites, such as the short-chain fatty acid (SCFA) butyrate [24]. Claesson and colleagues showed that cognitive performance was worse in those with reduced gut microbial diversity [25]. Moreover, Vogt et al. revealed that those with AD had lower gut microbial diversity compared with controls [26]. Lastly, apolipoprotein E4 carriers, who are at high risk for AD, had lower abundances of butyrate-producing gut microbes [27]. Also, IWL increases the abundance of beneficial gut microbial species, such as *Akkermansia muciniphila* [28], which was shown to be positively correlated with cognitive performance in older, obese African American adults [29]. Furthermore, IWL may promote increased fecal butyrate [30], an SCFA that is protective in the pathophysiology of AD [31]. Hence, the effect of a Med Diet and IWL on cognitive health may be mediated by the gut microbiome.

To our knowledge, no study has assessed the impact of a Med Diet lifestyle intervention with or without IWL on the gut microbiota in African American adults with obesity or determined whether these gut microbiota-associated changes, should they exist, correlate with changes in cognitive performance.

To address this gap, our group performed a post hoc analysis of the Building Research in Diet and Cognition (BRIDGE) trial, a randomized controlled lifestyle intervention trial that tested the effect of a Med Diet with or without IWL, compared with a usual diet condition, on cognitive and cardiometabolic health among older, predominately African American adults with obesity. In this post hoc analysis, we assessed whether participation in the interventions was associated with changes in gut microbiota structure, abundance, and butyrate production potential and whether changes in these gut microbial measures were related to changes in cognitive performance.

## 2. Materials and Methods

### 2.1. Study Design

Data and samples for the current study were collected from the BRIDGE trial. The BRIDGE Trial is registered at ClinicalTrials.gov (accessed on 1 June 2023) (NCT03129048). The intervention design and post-intervention analyses of the BRIDGE trial have been published [32,33]. Briefly, 185 participants were randomized in a 2:2:1 allocation ratio to a lifestyle program adopting a Med Diet (MedA); a lifestyle program adopting a Med Diet while restricting calories to generate IWL (MedWL); or a usual diet, in which participants were asked to maintain their habitual diet and physical activity level throughout the 14-month intervention. Participants were recruited in three separate cohorts of ~60 participants each between 2017 and 2019. Cardiometabolic, cognitive, dietary, anthropometric, and lifestyle variables were assessed at baseline, 8 months (at the end of the intervention), and 14 months (6 months after the end of the intervention). Stool was collected only from the first two cohorts (*n* = 124) and only at baseline and eight months. The eligibility criteria for the BRIDGE trial are defined elsewhere [33]. Briefly, the inclusion criteria included a BMI of 30–50 kg/m^2^, an age of 55–85 years, and a score of ≥19 on the Montreal Cognitive Assessment (MoCA) [34]. The MoCA was chosen over the Mini Mental State Exam (MMSE) because the MoCA is more sensitive for detecting those with MCI [35], a group at high risk for AD [1] that would, consequently, stand to benefit greatly from the intervention. The exclusion criteria included uncontrolled diabetes (hemoglobin A1c > 9.0), the use of an assistive walking device, adherence to a Med Diet (defined as a score of >6 on a 13-point Med Diet Adherence scale [36]), and psychiatric disorders.

For the current analysis, the gut microbiomes of participants who were in the MedWL and MedA groups, provided stool at baseline and eight months, and did not take oral or intravenous antibiotics within the six weeks prior to both data collection periods (*n* = 68) were analyzed. Given the exploratory nature of this post hoc analysis, a power analysis was not conducted. Those taking antibiotics were excluded because antibiotics substantially perturb the gut microbiome [37]. The usual diet group was also excluded (*n* = 11). Three of the control participants had lost ≥5% of their body weight at 8 months, and for this analysis, they would not have served as reliable comparators. Lastly, two individuals were removed due to labeling errors, leaving *n* = 66 individuals with evaluable data. The University of Illinois Chicago (UIC) Institutional Review Board reviewed and approved the study procedures (IRB #2016-0258).

#### Intervention Description

The interventional design and content have been described elsewhere [33]. Briefly, the MedA and MedWL groups were led by separate research dietitians. The dietitians met one-on-one with each participant before group classes began to introduce the tenets of a Med Diet. After this initial meeting, there were 25 one-hour group sessions held over eight months in which the participants learned via didactic and hands-on activities how to adopt and maintain a Med Diet pattern either with (MedWL) or without (MedA) instructions to lose weight via caloric restriction. Accordingly, dietary recommendations that promoted weight maintenance were implemented in the MedA arm, and recommendations that would lead to ~7% IWL at eight months via caloric restriction were implemented in the MedWL arm. To further promote IWL in the MedWL arm, this group (and this group only) was offered a half hour of supervised group exercise after each diet session.

### 2.2. Measures

#### 2.2.1. Stool and Blood Collection

Participants provided a fasting blood sample (≥8 h) through venipuncture. Blood was centrifuged at 3000 RPM at 4 °C to isolate the serum and plasma, which was then transported on dry ice to a −80 °C freezer and stored until analysis. Participants collected stool at home 24–48 h before each data collection period and stored it in a home refrigerator until the study visit. Participants transported the stool with an ice pack to each data collection visit. As some gut bacterial taxa may be affected by storage time and temperature [38], 60–72 h elapsed between the time of stool production and the time the stool sample was stored at −80°C to standardize the holding time across samples.

#### 2.2.2. DNA Extraction, Library Prep, and Sequencing

The Genome Research Core (GRC) at UIC extracted genomic DNA from stool samples with the DNeasy PowerSoil Kit (Qiagen, Valencia, CA, USA) and a bead-beating procedure using the FastPrep-24 System (MP Biomedicals, Irvine, CA, USA). The V4 region of the 16S rRNA gene of the previously extracted genomic DNA was amplified using the polymerase chain reaction (PCR) in a two-stage targeted amplicon sequencing (TAS) protocol [39]. In stage one, primers CS1_515F and CS2_806R from the Earth Microbiome Project and MyTaq HS 2X Master Mix (Bioline) were added to the PCR to target the V4 region [40]. In stage two, a unique 10-base barcode and MyTaq were added (Fluidigm, South San Francisco, CA, USA; Item# 100-4876). To equalize the sequencing depth between samples [41], equal volumes from each sample were pooled with an EpMotion5075 liquid-handling robot (Eppendorf, Hamburg, Germany) and then filtered to remove DNA less than 300 bp using an AMPure XP cleanup protocol (0.6×, *vol*/*vol*; Agencourt, Beckmann-Coulter). An Illumina MiniSeq flow cell with a 20% phiX spike-in was then loaded with the filtered DNA. If the number of reads between samples was unbalanced, then the samples were re-pooled in quantities inversely proportional to their original number of reads, purified with AMPure XP cleanup, loaded onto a Miniseq flow cell with a 20% phiX spike-in, and then re-sequenced to generate 2 × 153 paired-end reads. Negative controls were added during DNA extraction and PCR amplification to detect possible contamination.

#### 2.2.3. Basic Data Processing with DADA2

PEAR v0.9.6 was used to merge forward and reverse reads [42], resulting in 9,344,697 merged reads. Reads were subsequently removed with cutadapt v1.18 if they did not meet the quality threshold of *p* = 0.01, if they had internal ambiguous nucleotides, if they lacked a primer sequence, or if they were less than 225 base pairs [43]. Cutadapt was also used to remove primer sequences and ambiguous nucleotides from the ends of reads. The USEARCH v8.1.1861 algorithm was used to remove chimeric sequences, utilizing the Silva database (v132) for comparisons [44,45]. After these quality control measures were undertaken, a total of 693,306 reads were removed, leaving 8,651,391 reads available for analysis. The amplicon sequence variants (ASVs) were identified and annotated with a taxonomic assignment via the Naïve Bayesian classifier and the Silva v132 training set in DADA2 (v1.18) [46].

#### 2.2.4. Differential Analysis of Microbial Taxa

The software package edgeR was used to model the abundance of each taxon as a function of the independent variables, including the change in Med Diet adherence score, percent weight change, and change in each of the three cognitive domain scores [47]. Prior to analysis, chloroplast or mitochondrial DNA sequences were filtered out, and taxa with less than 100 counts summed across all samples or present in less than 25% of the samples were removed. Data were normalized as counts per million and modeled by a negative binomial generalized linear model that included all independent variables listed above as predictors and controlled for the subject. The likelihood ratio test was used to provide *p*-values, which were transformed into q-values with the Benjamini–Hochberg false discovery rate (FDR) correction [48]. The statistical significance of taxa was set at q < 0.05.

#### 2.2.5. Alpha Diversity Analyses

Prior to alpha diversity analysis, data were rarefied to a depth of 30,000 counts per sample. Thereafter, the vegan library in R was used to compute Shannon indices using default parameters [49,50]. The Shannon indices were modeled as a function of each independent variable while controlling for the subject ID. A generalized linear model (GLM) assuming a Gaussian distribution was utilized, and the likelihood ratio test was used to determine the statistical significance of the models. To create figures, ggplot in R was used [51].

#### 2.2.6. Beta Diversity/Dissimilarity Analyses

Prior to the beta-diversity analysis, normalized data were square-root transformed. Thereafter, the vegan library in R was used to compute Bray–Curtis indices using default parameters [49,52]. These indices were modeled as a function of each independent variable while controlling for the subject ID. The indices were scaled via non-metric multidimensional scaling (NMDS) and plotted with ggplot in R [51]. Significance tests were carried out using the ADONIS test in the vegan package in R.

#### 2.2.7. Butyryl-CoA CoA-Transferase Gene (*BcoA*)

*BcoA* encodes the *BcoA* enzyme, which is involved in the final step of microbial butyrate production [53]. *BcoA* abundance was measured with real-time PCR (rt-PCR). Integrated DNA Technologies (IDT, WI) created the BCoATscrF and BCoATscrR primers for *BcoA* as well as the synthetic DNA standard of 530 base pairs (bp) following the method of Louis et al. [54]. After optimizing primer efficiency, the microbial DNA extracted for sequencing was amplified in triplicate by the ViiA7 rt-PCR System (Applied Biosystems, Foster City, CA, USA) using Fast SYBR^®^ Green Master Mix (Applied Biosystems, Foster City, CA, USA) and the following parameters: two minutes at 50 °C; two minutes at 95 °C; and 40 cycles of one second at 95 °C, 20 s at 58 °C, and 30 s at 72 °C each with data acquisition at 72 °C. The melt curve data from primer optimization generated ambiguous results, and thus a 2% agarose E-Gel (Life Technologies, Carlsbad, CA, USA) was used to verify that there was a PCR product for each sample. The GRC at UIC performed the *BcoA* analysis described above.

Systemic Inflammation. High-sensitivity C-reactive protein (hs-CRP) was chosen as a marker of systemic inflammation; it is a potential risk factor for ADx [55] and is associated with the gut microbiome [56]. Quest Diagnostics (Wood Dale, IL, USA) used nephelometry to measure hs-CRP (lower limit of quantitation = 0.2 mg/L).

Glucose. Quest Diagnostics (Wood Dale, IL, USA) measured Hemoglobin A1c (HbA1c) using immunoturbidity (CV% = 1.09) and measured fasting serum glucose using spectrophotometry (CV% = 1.506). These were measured because diabetes is a risk factor for ADx [5] and is associated with gut microbial diversity [57].

Blood Pressure. An automated blood pressure monitor (Omron HEM-907 (Lake Forest, IL, USA)) was utilized to measure the diastolic and systolic blood pressure in duplicate with the participant in a calm environment and seated. Because midlife hypertension increases the risk for ADx [5] and is associated with the gut microbiome [58], blood pressure was reported.

Anthropometrics and Body Composition. A digital scale (Tanita, Arlington Heights, IL, USA) and a fixed stadiometer (Seca, Birmingham, UK) were used to measure weight and height, respectively. Weight was measured to the nearest 0.1 kg and height was measured to the nearest 0.1 cm in duplicate. BMI was reported as kg/m^2^. Body composition was calculated using dual-energy X-ray absorptiometry (DXA) with a Lunar iDXA machine (GE Healthcare, Chicago, IL, USA). Visceral adipose tissue (VAT) was calculated by the DXA machine and reported here, as it is negatively associated with cognitive performance [59] and is related to the gut microbiome [60].

Dietary Intake. The semi-quantitative Harvard Food Frequency Questionnaire (HFFQ) was administered by trained staff at baseline to capture habitual diet over the past year and then once again post-intervention to capture dietary intake during the intervention only (i.e., during the 8 months prior) [61]. The HFFQ measures the frequency of consumption and serving sizes of 131 foods/beverages and was processed by the Channing Lab at Harvard University. It has been validated with 24 h diet recall data and provides a more relevant measure of habitual diet than a 24 h recall [62].

Med Diet Adherence. A Med Diet adherence score (MED score) was derived from the HFFQ results. The score was originally created by Panagiotakos et al. [63] and then adapted by Tangney et al. [64] for an urban Midwestern population. To generate the score, foods on the HFFQ were classified into one of 11 categories: (1) non-refined grains, (2) potatoes, (3) fruit, (4) vegetables, (5) legumes and nuts, (6) fish, (7) olive oil, (8) red meat and processed meat, (9) poultry, (10) full-fat dairy products, and (11) alcohol. For each of these categories, a score of 0 to 5 was assigned, with 0 being minimal adherence and 5 being maximal adherence. For categories 1–7, the following scale was used: 0 = never, 1 = rare, 2 = frequent, 3 = very frequent, 4 = weekly, and 5 = daily consumption. For categories 8–10, the reverse of this scale was used, i.e., 0 = daily consumption, 1 = weekly consumption, etc. For alcohol, category 11, a score of 0 was assigned to the lowest and highest levels of consumption, with any other level varying directly with the score. Participants could achieve a MED score ranging from 0 to 55 points.

Cognitive Performance. Research assistants trained and certified by a qualified, licensed neuropsychologist administered an hour-long neuropsychological testing battery to participants at baseline and post-intervention. As recommended by the National Institute of Neurological Disorders and Stroke–Canadian Stroke Network’s Neuropsychology working group, cognitive processes were grouped into three domains: (1) attention and information processing (AIP), (2) executive function (EF), and (3) learning, memory, and recognition (LMR) [34]. AIP was evaluated with Digit Span Forward and Digit Symbol Coding subtests of the Wechsler Adult Intelligence Scale (WAIS-IV) [65], Stroop word and color tests [66], and Trail Making Test (TMT) Part A [67]. EF was measured using the verbal fluency letter [66,68], the Digit Span Backward and Sequencing subtests of the WAIS-IV, TMT Part B, and Stroop Color–Word Interference. LMR was assessed with total learning over 5 word-learning trials, delayed free recall, and recognition from the California Verbal Learning Test Second Edition (CVLT II) [69]. A composite score for each domain was created by generating a z-score for performance on each domain-specific test and taking the average of those z-scores. The z-scores were multiplied by −1 when higher scores on domain-specific tests represented worse performance.

Physical Activity. To obtain an objective measure of physical activity [70], participants wore an accelerometer (ActigraphGT3X + monitor, Pensacola Florida) on their non-dominant wrist for at least 4 days, 10 h per day. Sedentary time was calculated via ActiLife v6.13.4 software (ActiGraph, Pensacola, FL, USA) and defined as <2000 counts per minute [71]. Sedentary time was calculated as hours per valid day. 

Socio-demographic and Health Variables. At baseline, participants self-reported educational attainment, race and ethnicity, smoking status, and household income per year. Depressive symptoms were measured with the Center for Epidemiological Studies—Depression (CES-D) questionnaire, which is also used to identify those at high-risk for clinical depression [72].

Medication and Supplement Use. At baseline and post-intervention, the research team recorded medications and supplements that had been consumed in the 30 days prior to each data collection visit, along with frequency of use and dosage. The medications and supplements reported here are those demonstrated to have large, replicated effects on the gut microbiome structure or composition (i.e., osmotic laxatives, statins, proton pump inhibitors, metformin, aspirin [73], fiber supplements [74], probiotics [75], and prebiotics [76]) and that may introduce unwanted variability in the gut microbiome results. Anti-inflammatory medication usage was reported to aid in the interpretation of any anti-inflammatory effect of diet or IWL and its potential association with cognition or the gut microbiome.

#### 2.2.8. Statistical Analysis

To determine the significance of differences between groups for the change over time of normal and continuous variables, a linear mixed-effects model was used (SAS procedure MIXED). Continuous variables were log-transformed to achieve normality, if possible. To determine the significance of differences between groups for the changes over time of nominal variables, generalized linear mixed models were used (SAS procedure GLIMMIX). In general, the significance of between-group differences at each time point and within-group differences for continuous and nominal variables was determined with *t*-tests using the lsmeans statement within the MIXED (for continuous variables) and GLIMMIX (for nominal variables) procedures. The significance of between-group differences for the change over time of any variable was determined with an *F*-test of the visit*variable interaction. Furthermore, the significance of within-group changes over time was adjusted with the Tukey–Kramer method, as these within-group changes were estimated from the visit*group interaction. In all models, the cohort, age at randomization, and MoCA score were controlled for. SAS 9.4 (SAS Institute Inc., Gary, NC, USA) was used for the statistical analysis. Significance was set at an adjusted *p*-value of <0.05.

#### 2.2.9. Mediation Analysis

The mediation analysis was conducted with the CAUSALMED procedure in SAS, a procedure that uses the regression adjustment method and generalized linear models to determine the significance and strength of the mediating effect. This was performed to explore potential interactions between the main variables of interest to inform future work. Specifically, we explored the mediating effect of post-intervention alpha diversity on the relationship between post-intervention LMR scores and post-intervention MED scores, controlling for baseline alpha diversity, LMR scores, MED scores, cohort, age, and MoCA score. Other significant covariates, including potential confounders, were further adjusted for more accurate analysis.

## 3. Results

Table 1 provides baseline characteristics of participants as well as post-intervention results. Participants were predominantly African American females. Because subsamples of those randomized to MedA and MedWL were used, there was some unbalance between the groups at baseline. Usage of gut microbiome-altering and anti-inflammatory medications and fasting glucose were significantly different at baseline between groups. Weight decreased significantly from baseline in both groups and by similar amounts: −3.0 kg in MedA and −4.4 kg in MedWL (*p* = 0.13 for visit*group interaction). The MED score increased significantly in both groups by similar amounts: 5.7 points in MedA and 5.9 points in MedWL (*p* = 0.9 for visit*group interaction).

**Figure 1:**
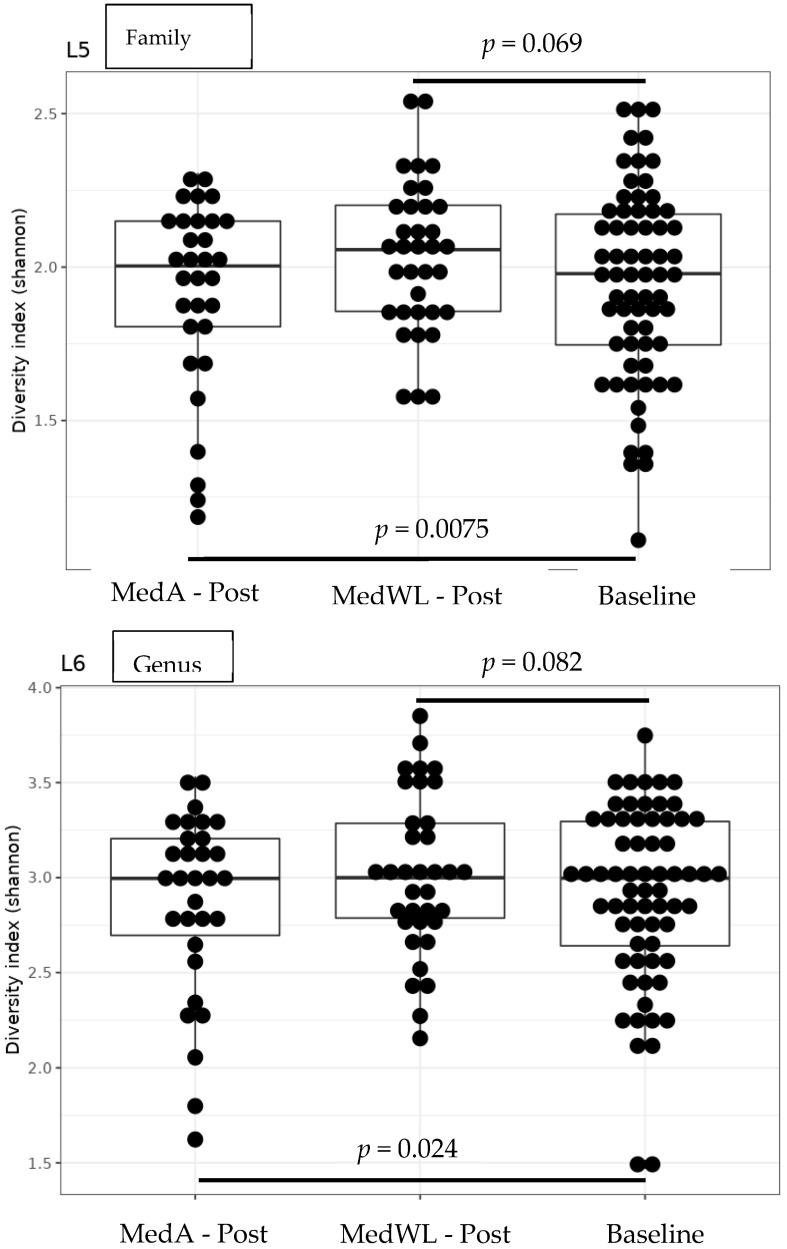
Box plots of Shannon Diversity at the family and genus levels. Baseline represents baseline values from both groups. MedA—post and MedWL—post are post-intervention values.

**Figure 2:**
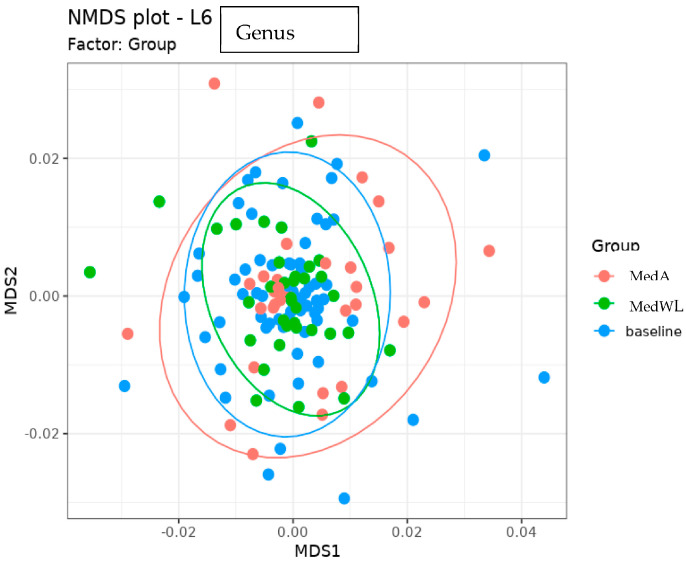
NMDS plot showing baseline Bray−Curtis beta−diversity values for all participants versus post-intervention beta-diversity values for MedA and MedWL.

Gut Microbiome. Group (MedA and MedWL) was significant at the family and genus levels for the models examining alpha diversity (*p* = 0.0075 and *p* = 0.024, respectively) (Figure 1). Group, however, was not significantly associated with beta diversity at any taxonomic level (Figure 2) nor with any taxon abundance. Similarly, there were no significant within-group changes in beta diversity or taxa abundances. Lastly, the change in *BcoA* abundance was not significantly associated with group assignment, but prior to adjusting for multiple comparisons, there was a significant decrease in *BcoA* abundance in the MedWL group only (Table 1). However, *BcoA* abundance was not significantly associated with IWL in a mixed linear model (*p* = 0.11).

**Figure 3:**
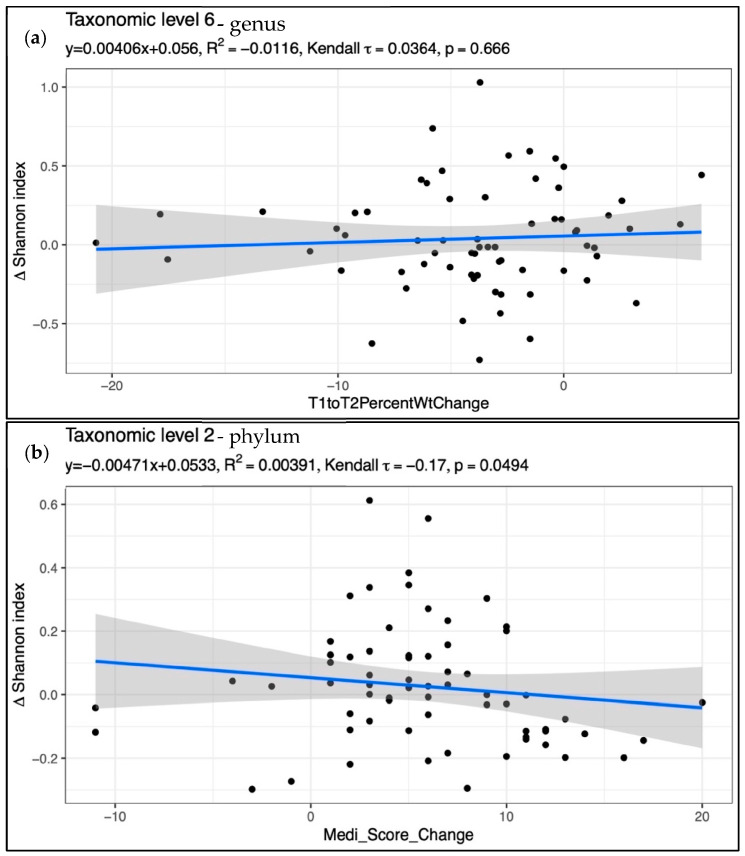
Scatter plot with a linear regression overlay comparing change in Shannon index with percent weight change (**a**) and MED score change (**b**). The p-value is for the Kendall tau statistic.

To explore whether the MED score change and IWL were related to changes in the gut microbiome, the MedA and MedWL groups were combined into one group, and alpha diversity, beta diversity, and taxa as a function of MED score and IWL were modeled. The change in MED score was significantly associated with alpha diversity, but only at the phylum level and in an unexpected direction, with an increased MED score associated with decreased alpha diversity (*p* = 0.049) (Figure 3b). IWL was not associated with alpha diversity, beta diversity, or any taxon abundance.

**Figure 4:**
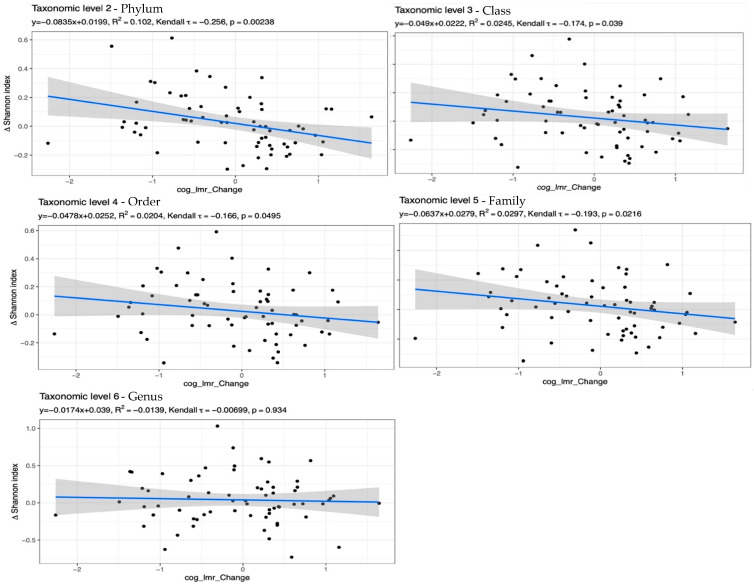
Scatter plots with linear regression overlays illustrating the relationship between change in the Shannon index over time and change in LMR scores over time. The *p*-value relates to the Kendall tau statistic.

Regarding cognitive performance, an increase in LMR score over time was associated with a decrease in alpha diversity at the phylum, class, order, and family taxonomic levels (Figure 4). EF and AIP scores were not associated with alpha diversity or beta diversity at any taxonomic level, with any taxon abundance, or with *BcoA* abundance.

### Mediation Analysis

Because phylum alpha diversity was associated with both the LMR score and MED score, the significance of phylum alpha diversity as a mediator of the relationship between the LMR score and the MED score was evaluated. The results indicated that phylum alpha diversity was not a significant mediator of the LMR/MED score relationship (IE = −0.00029, *p* = 0.49) (Table 2).

## 4. Discussion

In this post hoc analysis of an eight-month Med Diet lifestyle intervention with or without IWL among older, predominately African American, female adults with obesity, there was little effect of the intervention on the gut microbiome. There was a group effect for the change in alpha diversity, but there was no group effect for the change in either beta diversity or taxa abundance. Unexpectedly, an increase in the MED score and LMR score over the course of the intervention was associated with a decrease in alpha diversity.

The minimal effect of the interventions on the gut microbiome coincides with several prior studies showing that Med Diet interventions with or without IWL induce minor to no changes in gut microbiome diversity and composition [78,79,80,81,82,83,84,85,86,87,88,89,90,91,92,93,94,95]. However, several studies have shown an effect. Those that have demonstrated an effect either utilized a run-in period to exclude participants who may not adhere to the diet [88], provided all study foods [82,85,89], induced greater weight loss (i.e., mean of 8 kg) in participants [80], or increased the Med Diet adherence score by about 30% of the score’s range [81,88]. The significant effect of these studies on the gut microbiome may also have been due to larger sample sizes (i.e., 90–343 participants), longer durations (i.e., 12 months) [81], and greater changes in MED score [81] and weight [80]. In the current study, few study foods were provided, and there was no run-in period. Furthermore, IWL was lower (i.e., ~4 kg), and the MED score did not increase as much (i.e., 10% of the score’s range).

Improvements in LMR scores were significantly associated with decreases in alpha diversity at all taxonomic levels except the genus level. These findings should be interpreted with caution for several reasons. First, the mediation analysis revealed that the unexpected negative correlation between the LMR score and phylum alpha diversity was non-significant when controlling for MED score. Furthermore, the linear regression model revealed that change in LMR score accounted for less than 3% of the variation in the Shannon Index at the class, order, and family levels. Additionally, the Kendall tau coefficient for class, order, and family levels indicated a weak negative correlation between LMR scores and the Shannon index. Lastly, previous studies have reported that cognitive performance is significantly and positively correlated with higher gut microbial diversity, not lower. For example, Claesson and colleagues showed that cognitive performance was worse in those with lower gut microbial diversity [25]. More prospective studies and clinical trials should be conducted to confirm these results.

Lastly, *BcoA* abundance, a proxy for microbial butyrate production, was not influenced by the interventions and was not significantly associated with MED score, IWL, or cognition. No other studies have reported on the effect of a Med Diet with or without IWL on *BcoA* abundance, yet the results of a similar study contrasted with the results of this study [30]. In that study, *BcoA* increased after 3 months of an IWL trial among participants on a mildly hypocaloric diet that followed the national dietary guidelines of Germany, which promote many of the same foods as the Med Diet, including fruit, vegetables, legumes, nuts, and seeds [30]. More research is needed to understand how a Med Diet with or without IWL affects *BcoA* and microbial butyrate production.

This study has several strengths. To our knowledge, this is the first study to explore the impact of a Med Diet with or without IWL on the gut microbiome in African American adults. This group faces a disproportionate burden of dementia compared with several other groups [96]. Thus, including this high-risk group in research aiming to prevent or reduce dementia incidence is timely and important. Another strength is the measurement of several variables that may have unduly perturbed the gut microbiome during the intervention, such as changes in the utilization of gut microbiota-altering medications and supplements, including anti-, pre-, and probiotics. Lastly, the comprehensive cognitive evaluation allowed for an investigation of the cognitive domains previously shown to improve as a result of a Med Diet [19,97] and IWL [98,99].

This study has several limitations. First, weight loss in the MedA group and the relatively small sample size may have made it difficult to detect between-group differences. Second, as this was a post hoc analysis, randomization was lost. This lessened the ability to determine the individual effects of the MedA and MedWL arms. Third, because individuals who took antibiotics or who were unable to provide a stool sample were excluded, the generalizability of the results are reduced. Fourth, dietary intake was self-reported, which may have caused participants to provide socially desirable responses, leading to the misreporting of diet. Another limitation was the use of the MED score, which was not designed to capture dietary diversity, as it coalesces all types of a particular food into one group. However, the diversity of foods has been shown to explain variation in the gut microbiome [24]. Thus, the MED score may have been limited in its ability to relate to changes in the gut microbiome.

## 5. Conclusions

In conclusion, a Med Diet with or without IWL did not alter gut microbial diversity, composition, or function even though statistically significant improvements in Med Diet adherence and significant reductions in weight were observed. Future gut microbiome studies involving a Med Diet and IWL intervention in African American adults should consider using a larger sample size and sampling at 3 or 4 months after baseline to more fully capture the dynamics of the gut microbiome. Such studies would help confirm whether and how the gut microbiome mediates the effect of a Med Diet and IWL on cognitive performance and dementia risk.

## Figures and Tables

**Table 1:** Participant characteristics at baseline and post-intervention.

Variable	MedA Baseline (*n* = 31)	MedWL Baseline (*n* = 35)	*p* ^d^	MedA Post (*n* = 31)	MedWL Post (*n* = 35)	*p* ^e^
Sociodemographic, Diet, and Physical Activity Variables						
Age, years (mean (SD))	65.9 (6.7)	65.3 (5.3)	0.59	N/A	N/A	N/A
Female (*n*,%)	28, 90%	29, 82%	0.38	N/A	N/A	N/A
African American (*n*, %)	29, 93.6%	33, 94.3%	0.81	N/A	N/A	N/A
Any college education (*n*, %)	30, 96.8%	32, 91.4%	0.32	N/A	N/A	N/A
College graduate (*n*, %)	16, 51.6%	19, 54.3%	0.89	N/A	N/A	N/A
<USD 40 K annual household income (*n*, %)	12, 40%	17, 50%	0.33	N/A	N/A	N/A
MED Score (range 0–55) (mean (SD))	32.9 (6.3)	32.2 (4.8)	0.56	38.6 ^##,^** (4.9)	38.1 ^##,^** (4.8)	0.9
Sedentary time (minutes/valid day) (mean (SD)) ^a^	877.8 (147.6)	893.3 (149.7)	0.62	870.1 (134.3)	889.7 (116.8)	0.9
Medication Use						
Using microbiome-altering medications/supplements (*n*, %) ^b^	10, 32.3%	21, 60%	0.036	14, 45.2%	19, 54.3%	0.19
Using anti-inflammatory medications (*n*, %) ^c^	5, 16%	15, 43%	0.032	5, 16.1%	16, 45.7%	0.88
Clinical Variables						
Weight (kg) (mean (SD))	97.8 (13.6)	101.1 (16.4)	0.47	94.8 (12.1) ^##,^*	96.7 (18.6) ^##,^**	0.13
BMI (kg/m^2^) (mean (SD))	35.9 (4.8)	37.7 (4.1)	0.13	34.8 (4.2) ^##,^**	36 (4.9) ^##,^**	0.18
Visceral fat (g)/Height (cm) (mean (SD))	8.8 (3.8)	10.1 (4.8)	0.63	7.8 (2.9) ^##,^*	9 (4.9) ^##,^*	0.68
Depression score from CES-D (mean (SD))	5.7 (5.7)	9 (6) [77]	0.068	4.3 (4.5)	8.7 (6.5)	0.18
Currently smoking (*n*, %)	2, 6.5%	2, 5.7%	0.53	2, 6.5%	2, 5.7%	1.0
Systolic blood pressure (mmHg) (mean (SD))	131.2 (17.6)	132.6 (18.1)	0.72	131.8 (17.0)	134 (20.4)	0.88
Diastolic blood pressure (mmHg) (mean (SD))	80.9 (12.6)	80.8 (8.3)	0.99	79.0 (10.0)	81.9 (11.9)	0.3
Type 2 diabetes (*n*, %)	8, 25.8%	6, 17.1%	0.48	7, 22.6%	5, 14.3%	0.98
Fasting serum glucose (mg/dL) (mean (SD))	93.4 (12.6)	100 (14.3)	0.026	97.5 (19.5)	97.4 (17.5)	0.08
Hemoglobin A1c (% total Hb) (mean (SD))	6.0 (0.7)	5.9 (0.8)	0.82	5.8 (0.8) ^#^	5.8 (0.7) ^#^	0.98
hs-CRP (mg/L) (mean (SD))	4.3 (6.3)	5.0 (6.6)	0.74	6.7 (7.6)	6.4 (5.2)	0.97
Microbiome-Related Variables						
*BcoA* abundance (cycles until threshold) (Mean (SD))	25.6 (3.1)	26.4 (2.6)	0.33	25.8 (3.4)	27.4 (3.2) ^#^	0.1
Cognitive Variables						
MoCA Score (mean SD))	24.5 (2.6)	25.2 (2.8)	0.33	N/A	N/A	N/A
Attention (AIP) (mean (SD))	0.06 (0.5)	0.08 (0.7)	0.7	0.06 (0.6)	0.03 (0.7)	0.51
Executive Function (EF) (mean (SD))	−0.1 (0.5)	0.2 (0.6)	0.056	−0.2	0.2 (0.6)	0.81
Memory (LMR) (mean) (SD))	−0.01 (0.7)	0.03 (0.9)	0.89	−0.07 (0.8)	−0.05 (0.8)	0.9

This table shows baseline characteristics of participants by group. The statistical tests described below were used to determine significant differences within and between groups. * *p* < 0.05 for within-group difference, adjusted for multiple comparisons. ** *p* < 0.01 for within-group difference, adjusted for multiple comparisons. ^#^ *p* < 0.05 for within-group difference, not adjusted for multiple comparisons. ^##^ *p* < 0.01 for within-group difference, not adjusted for multiple comparisons. ^a^ Sedentary time was defined as <2000 counts per minute and calculated as total number of 1 min epochs of sedentary time divided by total number of valid days worn. ^b^ Gut microbiome-altering medications/supplements included fiber supplements, probiotics, prebiotics, osmotic laxatives, statins, proton pump inhibitors, metformin, and aspirin. ^c^ Anti-inflammatory medication comprised steroids, prednisone, aspirin, and NSAIDs. ^d^ *p*-Values for difference between groups at baseline. ^e^ *p*-values for difference between groups for change over time. AIP = attention and information processing; *BcoA* = Butyryl-CoA CoA-transferase Gene; BMI = body mass index; CES-D = Centers for Epidemiological Studies Depression; Hb = hemoglobin; hsCRP = high sensitivity C-reactive protein; K = thousand; LMR = learning, memory, and recognition; MedA = Med Diet alone; MedWL = Med Diet plus weight loss; MoCA = Montreal cognitive assessment; SD = standard deviation.

**Table 2:** Results of the analysis to identify the mediating effect of phylum alpha diversity on the relationship between MED score and the learning, memory, and recognition (LMR) score.

Mediation Analysis						
	Estimate	Standard Error	Wald 95% Confidence Limits		Z	Pr > |Z|
Total Effect (MED score + Phylum Alpha Diversity) on LMR Score	0.0454	0.0181	0.009827	0.08094	2.50	0.0124
Direct Effect (DE) (MED score) on LMR Score	0.0483	0.0185	0.01207	0.08453	2.61	0.0090
Indirect Effect (IE) (Phylum Alpha Diversity) on LMR Score	−0.00291	0.00422	−0.01119	0.005363	−0.69	0.4903

## Data Availability

Microbiome sequence data are publicly available at https://www.ncbi.nlm.nih.gov (accessed on 17 July 2023) in BioProject ID: PRJNA986915. Other select data is available upon request due to privacy restrictions.
